# Predictors of fluid responsiveness in patients with acute liver failure

**DOI:** 10.1186/cc9485

**Published:** 2011-03-11

**Authors:** VK Audimoolam, M McPhail, W Bernal, C Willars, JA Wendon, G Auzinger

**Affiliations:** 1 King's College Hospital, London, UK

## Introduction

Profound hemodynamic changes seen in acute liver failure (ALF) resemble those found in later stages of septic shock. Vasopressor support is frequently required and indiscriminate fluid resuscitation can worsen intracranial hypertension (ICH) and lung injury. Markers of preload dependency have thus far not been studied in this patient group and response to dynamic manoeuvres such as passive leg raising or end expiratory hold cannot be considered safe due to the high incidence of ICH.

## Methods

ALF patients admitted to a tertiary specialist ICU in vasoplegic shock, requiring multiorgan support including controlled mechanical ventilation, had their cardiac output monitored via transpulmonary thermodilution and pulse contour analysis (PiCCO). Markers of fluid responsiveness were compared between responders (CI ≥15%) and nonresponders to a colloid fluid challenge (5 ml/kg IBW). All patients had a transthoracic echocardiogram performed before and after fluid administration. The predictive capacity of stroke volume, pulse pressure variation (SVV, PPV) and respiratory change in peak aortic velocity ΔV peak for preload dependency was analyzed.

## Results

Twenty-six patients (mean age 40 (13), 15 male:11 female) were assessed, mean APACHE II 23 (4) and SOFA 15 (2). Changes in CI and SVI were closely correlated (*R *= 0.726, *P *< 0.001). There was no difference between those defined as responders using a cut-off value of CI or SVI of 10%. When using 15%, seven patients would have been classified differently. The intraclass correlation coefficient for CI and SVI change was 0.83 (0.62 to 0.92), confirmed using Pasing and Blakock regression (*A *= -0.278, -0.88 to 0.16, *B *= 1.26, 0.88 to 1.72), suggesting hemodynamic changes in both measures are interchangeable. Using a cut-off value of a change in CI of 15%, only PPV predicted fluid responsiveness (AUROC 0.79, 0.58 to 0.93, *P *= 0.005, cut-off >9%, sensitivity 75%, specificity 62%). SVV weakly predicted fluid responsiveness in this cohort (AUROC 0.73, 0.52 to 0.87, *P *= 0.005, cut-off >11%). While there was a trend toward reduction in ΔV peak (mean difference -3%, *P *= 0.080) this was not different between those defined as fluid responders by CI (repeated-measures ANOVA *P *= 0.124) and ΔV peak prior to fluid bolus did not predict a CI response (AUROC 0.637, 0.413 to 0.825, *P *= 0.322).

## Conclusions

Baseline PiCCO parameters predict fluid responsiveness but the respiratory variability in ΔV peak did not predict a CI response to fluid bolus in this cohort. PPV may be a more suitable PiCCO index for assessing fluid requirements in patients with ALF than SVV.

